# M2-type tumor-associated macrophages upregulated PD-L1 expression in cervical cancer via the PI3K/AKT pathway

**DOI:** 10.1186/s40001-024-01897-2

**Published:** 2024-07-05

**Authors:** Fan Guo, Weina Kong, Dewei Li, Gang Zhao, Miyessar Anwar, Feifei Xia, Yuanming Zhang, Cailing Ma, Xiumin Ma

**Affiliations:** 1 Department of Medical Laboratory Center, Tumor Hospital Affiliated to Xinjiang Medical University, State Key Laboratory of Pathogenesis, Prevention and Treatment of High Incidence Diseases in Central Asia, No 789 Suzhou Road, Urumqi, 830011 Xinjiang China; 2 Postdoctoral Research Workstation of Tumor Hospital Affiliated to Xinjiang Medical University, Urumqi, Xinjiang China; 3 Center of Respiratory and Critical Care Medicine, The People’s Hospital of the Xinjiang Uygur Autonomous Region, Urumqi, Xinjiang China; 4 Department of Blood Transfusion, Affiliated Traditional Chinese Medicine Hospital of Xinjiang Medical University, Urumqi, Xinjiang China; 5 Tumor Hospital Affiliated to Xinjiang Medical University, Urumqi, Xinjiang China; 6 Department of Gynecology, The First Affiliated Hospital of Xinjiang Medical University, State Key Laboratory of Pathogenesis, Prevention and Treatment of High Incidence Diseases in Central Asia, 137 Li Yu Shan South Road, Urumqi, 830054 Xinjiang China

**Keywords:** Cervical cancer, Tumor-associated macrophages (TAMs), Prognosis, Tumor microenvironment (TME)

## Abstract

**Background and purpose:**

PD-1/PD-L1 inhibitors have become a promising therapy. However, the response rate is lower than 30% in patients with cervical cancer (CC), which is related to immunosuppressive components in tumor microenvironment (TME). Tumor-associated macrophages (TAMs), as one of the most important immune cells, are involved in the formation of tumor suppressive microenvironment. Therefore, it will provide a theoretical basis for curative effect improvement about the regulatory mechanism of TAMs on PD-L1 expression.

**Methods:**

The clinical data and pathological tissues of CC patients were collected, and the expressions of PD-L1, CD68 and CD163 were detected by immunohistochemistry. Bioinformatics was used to analyze the macrophage subtypes involved in PD-L1 regulation. A co-culture model was established to observe the effects of TAMs on the morphology, migration and invasion function of CC cells, and the regulatory mechanism of TAMs on PD-L1.

**Results:**

PD-L1 expression on tumor cells could predict the poor prognosis of patients. And there was a strong correlation between PD-L1 expression with CD163^+^TAMs infiltration. Similarly, PD-L1 expression was associated with M1/M2-type TAMs infiltration in bioinformatics analysis. The results of cell co-culture showed that M1/M2-type TAMs could upregulate PD-L1 expression, especially M2-type TAMs may elevate the PD-L1 expression via PI3K/AKT pathway. Meanwhile, M1/M2-type TAMs can affect the morphological changes, and enhance migration and invasion abilities of CC cells.

**Conclusions:**

PD-L1 expression in tumor cells can be used as a prognostic factor and is closely related to CD163^+^TAMs infiltration. In addition, M2-type TAMs can upregulate PD-L1 expression in CC cells through PI3K/AKT pathway, enhance the migration and invasion capabilities, and affect the tumor progression.

## Introduction

Cervical cancer (CC), as a malignant tumor, is one of the leading causes of death in women, accounting for approximately 12% of female cancers [1, 2]. Despite improvements in the early diagnosis and treatment of CC, data from the World Health Organization showed that there were still 604,127 new cases and 341,831 deaths in 2020 [3]. It is well known that patients with early stage can be cured by surgery, and simultaneous radiotherapy and chemotherapy are the preferred treatment for patients with locally advanced CC. However, there is a high probability for recurrence and metastasis in patients with advanced CC, resulting in a median overall survival (OS) of only 16.8 months [4], which indicates that the therapeutic efficacy is limited. In China, the onset age of CC is getting younger, and Xinjiang is a region with a high incidence of CC [5]. Therefore, it is an urgent clinical problem to improve the therapeutic effect of CC patients.

Recently, an increasing number of studies have revealed the remarkable therapeutic effects of immunotherapy [6]. The programmed cell death factor 1 (PD-1) / programmed cell death ligand 1 (PD-L1) inhibitor, as classical immune checkpoint inhibitors (ICIs), have achieved good clinical outcomes in various solid tumors and are considered to be a promising tumor treatment strategy. Pembrolizumab (MK-3475), a PD-1 inhibitor, was initially studied in KEYNOTE-028. The 24 patients with PD-L1-positive advanced CC were enrolled, and the objective response rate (ORR) reached 17% [7]. KEYNOTE-158 is a further study of pembrolizumab. This cohort included 98 patients with recurrent or metastatic CC with an ORR of 12.2%, among which the ORR of PD-L1-positive CC patients reached 14.6% [8]. Therefore, based on the safety and antitumor effects of pembrolizumab in KEYNOTE-158 clinical trials, pembrolizumab has been officially approved by the US Food and Drug Administration for patients with advanced PD-L1-positive CC who experienced progression during or after chemotherapy. Although pembrolizumab improved the curative effect of patients with CC, the patient response rate was lower than 30% [9], considering to be associated with the immunosuppressive component in the tumor microenvironment (TME). Tumor-associated macrophages (TAMs), as one of the most important immune cells, are involved in the formation of tumor suppressive microenvironment. Hence, it will provide a new idea for curative effect improvement and combination therapy about the regulatory mechanism of TAMs on PD-L1 expression in CC.

TAMs, which refer to macrophages infiltrated in tumor tissues or distributed in the TME, are the most abundant in number of immune cell population that play a key role in the TME [10]. Currently, TAMs generally are divided into two main groups called classically activated macrophages (M1-type) and alternatively activated macrophages (M2-type) [11]. The two polarization states of TAMs are dynamically changing under the stimulation of different signals during the process of tumor occurrence and development. It is found that M1-type TAMs are mainly dominant groups in the initial stage of malignant tumor to improve the immune effect and exert antitumor effects. However, the expression of molecules such as interleukin-4 (IL-4), colony-stimulating factor 1 (CSF-1) and arginnse-1 (Arg-1) in the TME are increased with the development of tumor, which induce the transformation of M1-type TAMs into M2-type TAMs. So M2-type TAMs gradually occupy an epigenetic advantage, promoting tumor development and drug resistance [12, 13].

Previous studies have shown that the infiltration of TAMs in TME can elevate PD-L1 expression levels on a variety of tumor cells, promote the formation of tumor immunosuppressive microenvironment, and thus mediate the immune escape of tumor cells [14–16]. Therefore, TAMs are one of the regulatory factors for PD-L1 expression on tumor cells. In the mechanism study of Komura and Zhang et al., it was found that TAMs can upregulate the expression of PD-L1 in cancer cells by activating the phosphatidylinositol 3-kinase/protein kinase B (PI3K/AKT) signaling pathway [17, 18]. In CC, the expression of PD-L1 on tumor cells is highly correlated with the density of TAMs [19]. There is currently insufficient evidence to indicate whether the correlation between PD-L1 and TAMs is caused by TAMs regulating PD-L1. Moreover, the PI3K/AKT pathway regulates multiple cellular and molecular functions, which are crucial for tumor initiation, invasion and metastasis. Previous studies have shown that the PI3K/AKT pathway is often dysregulated in CC [20]. Therefore, the mechanism of TAMs activating PD-L1 expression on CC cells needs to be further explored.

Taken together, TAMs, as one of the important immune cells in TME, play a crucial role in the occurrence and development of tumors. TAMs can inhibit the recruitment and activation of T cells, regulate PD-L1 expression on tumor cells, and usually form a tumor suppressive microenvironment to affect the therapeutic efficacy of PD-1/PD-L1 inhibitors. There are two parts in our research. One part related to the study of clinical CC patient specimens and their survival rate. We performed immunohistochemistry (IHC) staining on CC patient tissues to observe the expression of TAMs and PD-L1, and analyzed the relationship between them. Moreover, the effect of PD-L1 on survival time of patients with CC was analyzed. And the other part related to the in vitro investigation of TAMs co-culturing with CC cell lines, aiming to explore the influence of TAMs on the biological behavior of CC cells and the regulatory mechanism of PD-L1. Meanwhile, bioinformatics was used to analyze the macrophage subtypes involved in PD-L1 regulation in the TCGA-CESC database. This is of great significance for screening the advantageous population of immunotherapy and guiding the clinical application of PD-1/PD-L1 inhibitors.

## Material and methods

### Collection of clinical data and patients samples

The clinical data and pathological embedded tissues of 98 patients with CC were collected from the Tumor Hospital Affiliated to Xinjiang Medical University after ethical review. Inclusion criteria: patients with CC diagnosed by pathological gold standard and had complete clinical data and paraffin pathological specimens. Exclusion criteria: history of other malignant tumors, autoimmune diseases, and during pregnancy or lactation. The survival status of 98 patients with CC in the study was followed up, mainly through medical record system inquiry and telephone follow-up. The  patients' OS was set as the time interval from the initial diagnosis of CC to the final death due to tumor factors or the last follow-up.

### IHC staining

IHC staining was used to analyze the expression of PD-L1, CD68 and CD163 in CC tissues. The procedures of IHC staining was as follows: baking tissue sections, dewaxing in xylene, hydrating in gradient alcohol, repairing antigen in microwave oven, inactivating endogenous peroxidase, blocking in goat serum, incubating antibodies, coloring, differentiating of hydrochloric acid ethanol and dehydrating, etc. The primary antibodies included rabbit anti-PD-L1 (ab213524, Abcam), mouse anti-CD68 (ab955, Abcam), and mouse anti-CD163 (TA506381, OriGene Technologies, Inc.). Evaluation of positive staining was performed by two independent pathologists who were blinded to the clinical data of patients.

### Bioinformatics analysis

The count matrix of transcriptome sequencing in TCGA-CESC database was downloaded by TCGAbiolinks package of R language. Gene set variation analysis (GSVA) package was used to analyze PD-L1 expression and the correlation of different subtypes of macrophages in TCGA-CESC database. Macrophages were divided into the following three types, and the gene signatures were as follows [21, 22]:

Macrophages: ANOS1, APOE, ATG7, BCAT1, CCL7, CD163, CD68, CD84, CHI3L1. CHIT1, CLEC5A, CTSK, CYBB, FN1, GM2A, GPC4, MARCO, MS4A4A, MSR1, RAI14, SCARB2, SGMS1.

M1-type macrophages: CCL18, CCL2, CCL3, CCL4, CCL5, CCR7, CD40, CD80, CD86, CSF2, CXCL10, CXCL11, CXCL16, CXCL8, CXCL9, FCGR1A, FCGR1B, FCGR2A, FCGR2B, FCGR3A, FCGR3B, IDO1, IFNG, IL12A, IL12B, IL15, IL18, IL1A, IL1B, IL1R1, IL23A, IL6, IRF5, KYNU, MMP9, PTX3, STAT1, TLR2, TLR4, TNF.

M2-type macrophages: ALOX15, ALOX5AP, ARG1, CCL1, CCL13, CCL17, CCL18, CCL22, CCL24, CCL4, CCR2, CCR3, CD14, CD163, CD200R1, CSF1, CXCL14, CXCR1, CXCR2, F13A1, FCER2, GATA3, HIF1a, ID3, IL10, IL13, IL17RB, IL1R1, IL4, IRF4, MARCO, MMP1, MMP12, MMP2, MRC1, MRC2, MSR1, NOS2, NR3C2, RGS1, SMAD2, SOCS1, SOCS3, SPP1, STAB1, STAT3, TGFB1, TGFBR2, TGM2.

### Cell culture

The human CC cell lines (SiHa and HeLa) and the human monocytic leukemia cell line (THP-1) were obtained from Procell Life Science&Technology Co., Ltd. Both SiHa and HeLa cell lines were adherent cells and were cultured in DMEM complete medium. THP-1 cell line was suspended cells and was cultured in 1640 complete medium. All cell lines were cultured in a cell incubator at 37 °C with 5% CO_2_. The induced differentiation of THP-1 cells was performed as follows: 1 × 10^6^ THP-1 cells were induced to adhere for 12 h with 320 nM PMA, then 100 ng/mL LPS and 20 ng/mL interferon-r (IFN-γ) were added for 48 h to obtain M1-type TAMs, and 20 ng/mL IL-4 and 20 ng/mL lL-13 were added for 48 h to obtain M2-type TAMs.

### Establishment of cell co-culture system

A non-contact co-culture system between CC cells and macrophages was established through Transwell cell chambers (0.4 μM) in vitro. The chamber was referred to as the upper chamber, which was inoculated THP-1 cell together with polarization cytokines to induce differentiating into M1/M2-type macrophage. The culture plate was called the lower chamber and inoculated with CC cells. The upper chambers contained induced macrophages were placed in the lower chambers seeded with CC cells, and co-cultured for 48 h. The 1640 complete medium was used for co-culture. It is worth noting that CC cells (SiHa and HeLa) were cultured using the 1640 complete medium for prior adaptation and stably passaged 3–5 generations for co-culture.

### Cell invasion and migration

Transwell 24-well cell culture plate (8 μM) was used in cell invasion. The Matrigel matrix glue was removed from – 80 °C and restored to liquid at 4 °C, then mixed with serum-free 1640 medium into the Transwell upper chamber until fully solidified. The serum-free suspension of 1 × 10^5^ CC cells was added to the upper chamber and the medium with 20% high concentration of fetal bovine serum was added to the lower chamber to attract cells. And they were placed in a 5%CO_2_ incubator at 37 °C. After 24 h, the upper chamber liquid was discarded, fixed with 4% paraformaldehyde for 20 min, and stained with 0.1% crystal violet solution for 20 min. The images could be taken for analysis after drying. Cell migration was performed without plating Matrigel matrix glue, and the remaining steps were the same as cell invasion.

### Flow cytometry

The cells were harvested and suspended in a PBS buffer containing 0.2% BSA. Centrifuge at 1500 r/min for 5 min, discard the supernatant and repeat 3 times. The cells were then stained with anti-PD-L1 PE (329,706, Biolegend, USA) for 30 min at 4 °C in the dark. The cells were detected by flow cytometry.

### Quantitative real-time PCR (qRT-PCR)

Total RNA was isolated from cells using Trizol according to the manufacturer’s instructions. The extracted RNA was converted into cDNA with 5 × gDNA eraser buffer, gDNA eraser, 5 × primescript buffer 2, primescript RT enzyme mix I, RT primer mix and RNase free dH_2_O. The qRT-PCR was performed in the QuantStudio 6 Real Time PCR Detection System machine in the presence of GAPDH, tumor necrosis factor α (TNF-α), IL-1β, transforming growing factor β (TGF-β), CD163, and PD-L1. The transcription level of target genes was measured and normalized to GAPDH expression. The following primer sequences were used: GAPDH, 5′-GGCAAATTCCATGGCACCGT-3′ (forward) and 5′-TGGACTCCACGACGTACTCA-3′ (reverse); TNF-α, 5′-TGCACTTTGGAGTGATCGGC-3′ (forward) and 5′-ACTCGGGGTTCGAGAAGATG-3′ (reverse); IL-1β, 5′-CAGTGGCAATGAGGATGA-3′ (forward) and 5′-TGTAGTGGTGGTCGGAGA-3′ (reverse); TGF-β, 5′-CGTGCTAATGGTGGAAACCC-3′ (forward) and 5′-TTCAGGTACCGCTTCTCGGA-3′ (reverse); CD163, 5′-GCGGGAGAGTGGAAGTGAAAG-3′ (forward) and 5′-GTTACAAATCACAGAGACCGCT-3′ (reverse); PD-L1, 5′-TATGGTGGTGCCGACTACAA-3′ (forward) and 5′-TGCTTGTCCAGATGACTTCG-3′ (reverse).

### Statistical analysis

GraphPad Prism 8.0 software was used for data statistical analysis. One-way analysis of variance was used to compare the mean values among groups of data with normal distribution. A nonparametric test was used to compare the mean values among groups of data with non-normal distribution. Counting data were descripted by percentage (%). And comparative analysis was performed using Pearson Chi-square test or Fisher exact test. Linear correlation or Spearman correlation analysis was used for correlation between two variables. Kaplan–Meier method was used to draw the patients' overall survival curve, and log-rank test was used to evaluate the difference of survival time between the two groups. A two-tailed test was used for all analyses. *P* < 0.05 was considered statistically significant.

## Results

### PD‑L1 expression and the infiltration of CD68^+^ and CD163^+^ macrophages

**Fig. 1:**
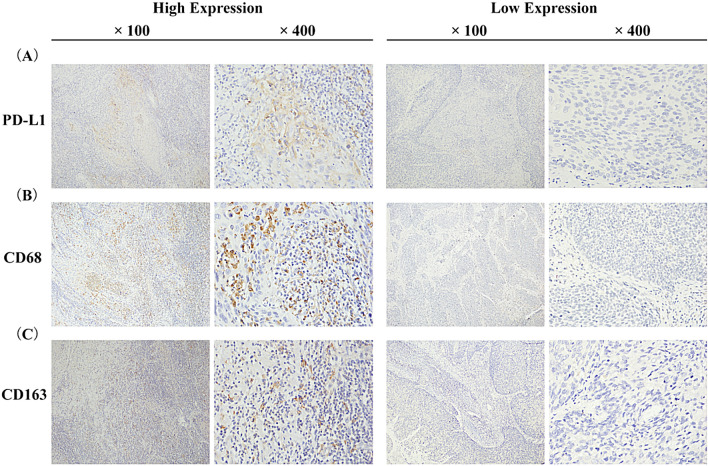
Immunohistochemistry staining for PD-L1, CD68^+^ and CD163^+^ macrophages in patients with cervical cancer. **A** PD-L1 expression. **B** CD68^+^ macrophages expression. **C** CD163^+^ macrophages expression. The left panel shows positive staining for PD-L1 and high macrophage density. The right panel shows negative staining for PD-L1 and low macrophage density

**Fig. 2:**
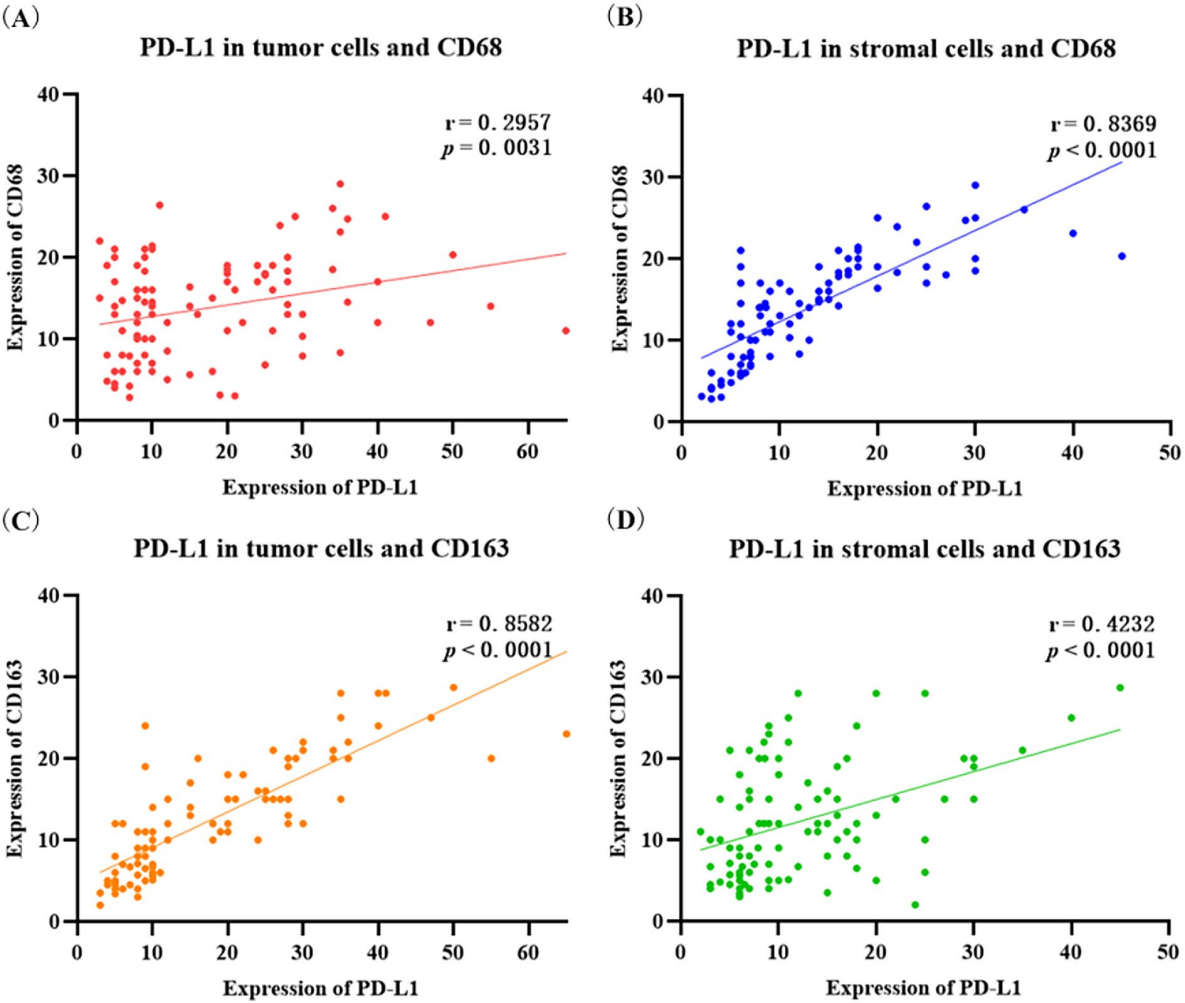
Correlation of PD-L1 expression and infiltration of CD68^+^ and CD163^+^ macrophages in patients with cervical cancer. **A** PD-L1 in tumor cells and CD68^+^ macrophages. **B** PD-L1 in stromal cells and CD68^+^ macrophages. **C** PD-L1 in tumor cells and CD163^+^ macrophages. **D** PD-L1 in stromal cells and CD163^+^ macrophages

Figure 1 shows the IHC staining images of PD-L1, CD68, and CD163. And IHC staining of PD-L1, CD68 and CD163 were performed in 98 patient tissues. The positive expression staining is brown. It was observed that PD-L1 was mainly expressed in the cytoplasm and membrane of tumor cells, and also partially expressed in stromal cells (Fig. 1A). Among 98 patients with CC (Table 1), 51 patients (52.04%) showed positive expression of PD-L1 on tumor cells, which was related to the patient's fertility history (*p* = 0.0042), abortion history (*p* = 0.0167), differentiation grade (*p* = 0.0381) and FIGO stage (*p* = 0.0440). PD-L1 was positively expressed in stromal cells in 45 patients (45.92%), which was related to fertility history (*p* = 0.0014), abortion history (*p* = 0.0100), tumor size (*p* = 0.0167) and FIGO stage (*p* = 0.0000). Moreover, the positive expression of CD68^+^ and CD163^+^ macrophages was mainly in the cell membrane, and the median distribution of intratumoral density in 98 CC samples was 14.0 and 12.0, respectively (Fig. 1B, C). Meanwhile, the infiltration of CD68^+^ and CD163^+^ macrophages in CC tissue was positively correlated with the expression of PD-L1 (Fig. 2). There was a weak correlation between the PD-L1 expression on tumor cells and the infiltration of CD68^+^ macrophages (*r* = 0.2957, *p* = 0.0031, Fig. 2A), and a strong correlation between PD-L1 expression on interstitial cells and the infiltration of CD68^+^ macrophages (*r* = 0.8369, *p* < 0.0001, Fig. 2B). PD-L1 expression on tumor cells was strongly correlated with the infiltration of CD163^+^ macrophages (*r* = 0.8582, *p* < 0.0001, Fig. 2C), and PD-L1 expression on interstitial cells was moderately related to the infiltration of CD163^+^ macrophages (*r* = 0.4232, *p* < 0.0001, Fig. 2D). These results indicated that the correlation between CD163 density and PD-L1 expression in tumor cells was stronger than that of CD68 density. And the correlation between CD163^+^ macrophages infiltration and PD-L1 expression in tumor cells was higher than that of stromal cells.

**Table 1:** Analysis of PD-L1 expression and clinical characteristics in patients with cervical cancer

Clinical characteristics	Cases (%)	PD-L1 expression on tumor cells		*P*	PD-L1 expression on mesenchymal cells		*P*
		Negative (%)	Positive (%)		Negative (%)	Positive (%)	
All cases	98	47 (47.96)	51 (52.04)		53 (54.08)	45 (45.92)	
*Age*				0.5329			0.5649
< 50	51 (52.04)	26 (50.98)	25 (49.02)		29 (56.86)	22 (43.14)	
≥ 50	47 (47.96)	21 (44.68)	26 (55.32)		24 (51.06)	23 (48.94)	
*Ethnicity*				0.7560			0.0936
Han	37 (37.76)	17 (45.95)	20 (54.05)		16 (43.24)	21 (56.76)	
Other minorities	61 (62.24)	30 (49.18)	31 (50.82)		37 (60.66)	24 (39.34)	
*HPV*				0.3097			0.3747
HPV 16	36 (36.73)	16 (44.44)	20 (55.56)		20 (55.56)	16 (44.44)	
HPV 18	29 (29.59)	11 (37.93)	18 (62.07)		14 (48.28)	15 (51.72)	
Other	20 (20.41)	11 (55.00)	9 (45.00)		12 (60.00)	8 (40.00)	
Negative	13 (13.27)	3 (23.08)	10 (76.92)		10 (76.92)	3 (23.08)	
*Fertility history*				0.0042			0.0014
0–2 times	52 (53.06)	32 (61.54)	20 (38.46)		36 (69.23)	16 (30.77)	
≥ 3 times	46 (46.94)	15 (32.61)	31 (67.39)		17 (36.96)	29 (63.04)	
*Abortion history*				0.0167			0.0100
Yes	42 (42.86)	26 (61.90)	16 (38.10)		29 (69.05)	13 (30.95)	
No	56 (57.14)	21 (37.50)	35 (62.50)		24 (42.86)	32 (57.14)	
*Tumor size*				0.5837			0.0167
≤ 4 cm	41 (41.84)	21 (51.22)	20 (48.78)		28 (68.29)	13 (31.71)	
> 4 cm	57 (58.16)	26 (45.61)	31 (54.39)		25 (43.86)	32 (56.14)	
*Differentiation*				0.0381			0.9337
Low	44 (44.90)	16 (36.36)	28 (63.64)		24 (54.55)	20 (45.45)	
Middle/high	54 (55.10)	31 (57.41)	23 (42.59)		29 (53.70)	25 (46.30)	
*Lymph node metastases*				0.1301			0.0972
Yes	77 (78.57)	40 (51.95)	37 (48.05)		45 (58.44)	32 (41.56)	
No	21 (21.43)	7 (33.33)	14 (66.67)		8 (38.10)	13 (61.90)	
*FIGO stage*				0.0440			0.0000
IA-IB	48 (48.98)	28 (58.33)	20 (41.67)		38 (79.17)	10 (20.83)	
≥ IIA	50 (51.02)	19 (38.00)	31 (62.00)		15 (30.00)	35 (70.00)

### Effect of PD-L1 expression on prognosis of patients with CC

**Fig. 3:**
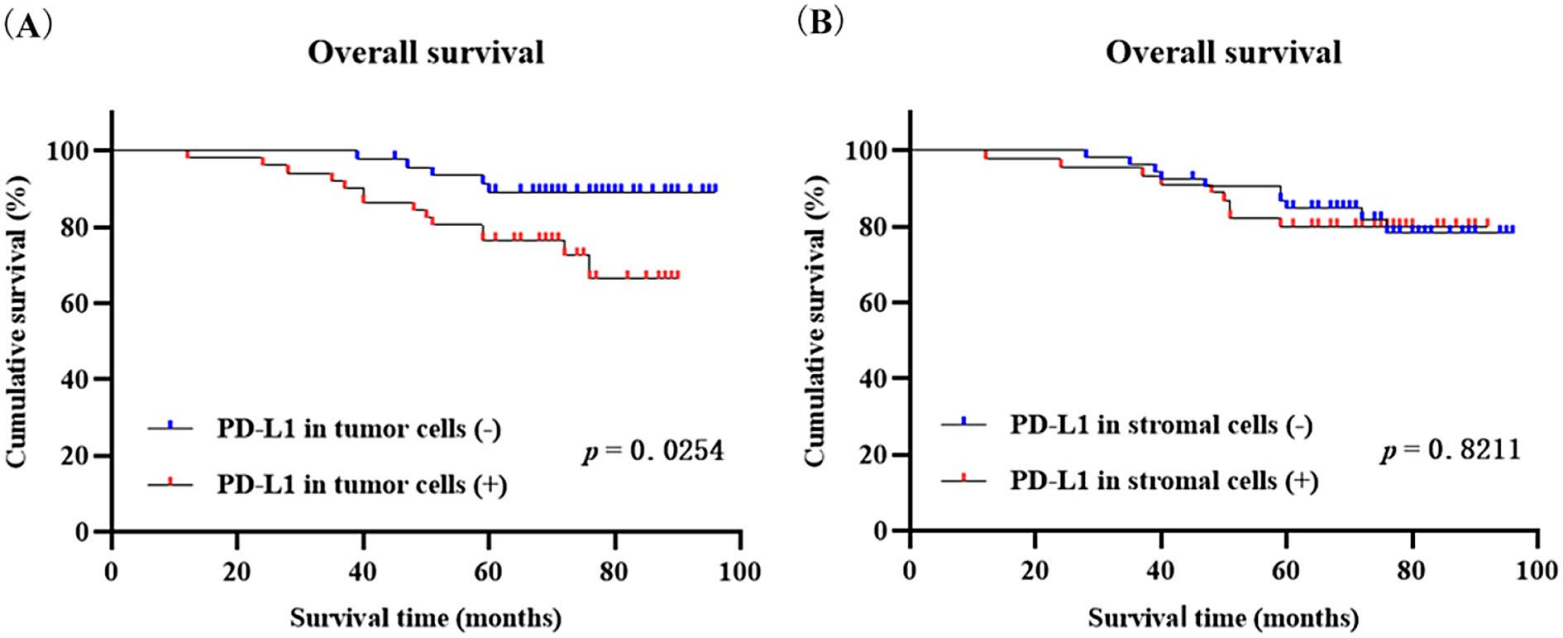
Kaplan–Meier overall survival curves of PD-L1 expression on tumor and stromal cells in patients with cervical cancer. **A** Kaplan–Meier overall survival curve of PD-L1 expression on tumor cells. **B** Kaplan–Meier overall survival curve of PD-L1 expression on stromal cells

19 cases of patients eventually died during the follow-up period of the 98 patients with CC. Since PD-L1 is mainly expressed on tumor cells and partly on stromal cells, we analyzed the influence of PD-L1 expression in different cells on patient prognosis. Log-rank test results showed that there was a statistically significant difference between the positive and negative expression of PD-L1 on tumor cells in CC tissue (*p* = 0.0254, Fig. 3A). However, there was no significant difference in OS between positive and negative expression of PD-L1 on stromal cells (*p* = 0.8211, Fig. 3B). In summary, the high expression of PD-L1 on tumor cells can predict poor prognosis of patients.

### Correlation between the expression of PD-L1 and M1/M2-type TAMs-related molecules in TCGA-CESC database

Through bioinformatics analysis of TCGA-CESC database, GSVA results showed that the expression of PD-L1 was positively related to pan-macrophages (*r* = 0.4279, *p* < 0.0001), M1-type macrophages (*r* = 0.5837, *p* < 0.0001) and M2-type macrophages (*r* = 0.4858, *p* < 0.0001).

**Fig. 4:**
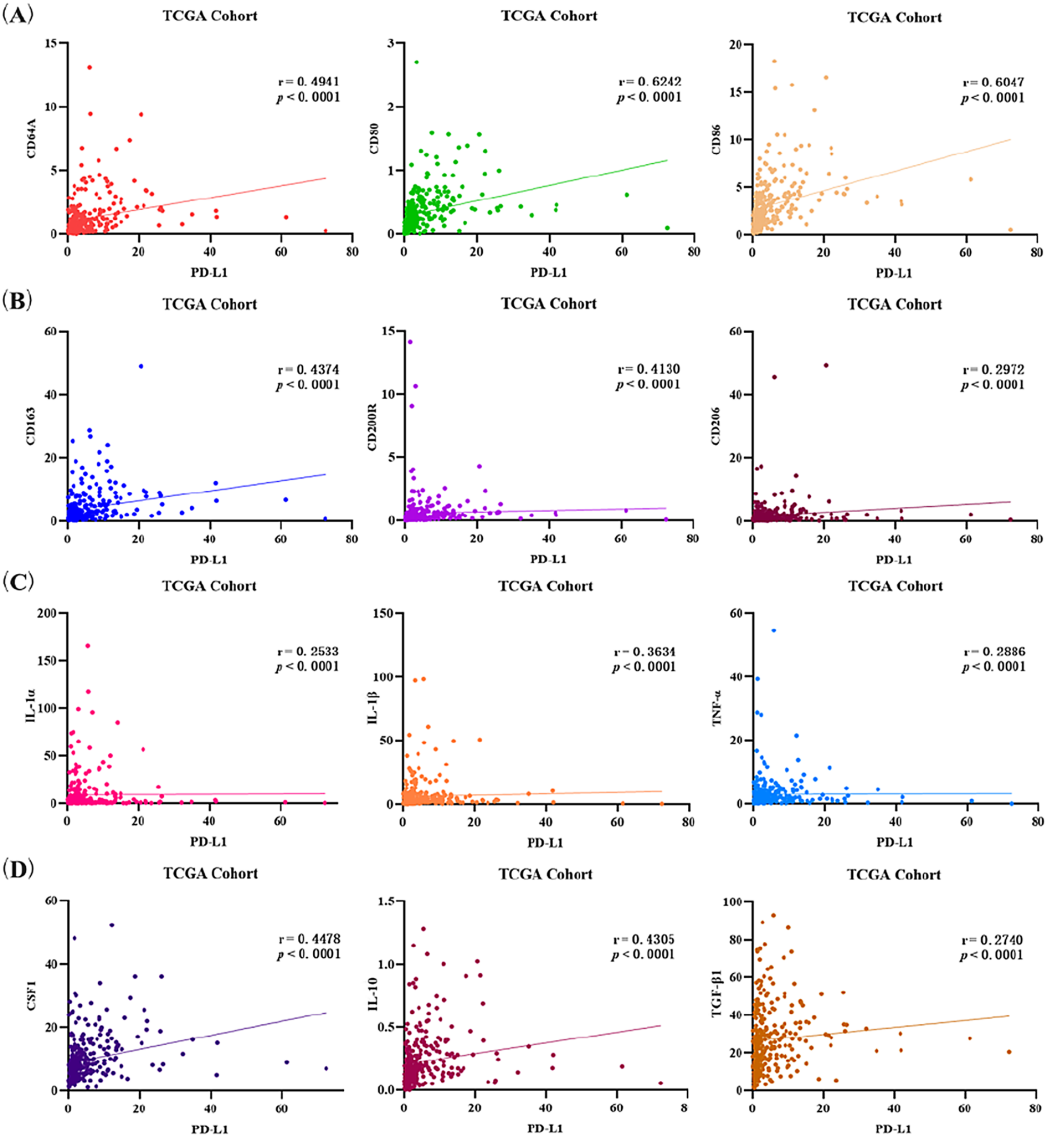
Correlation analysis between PD-L1 expression and TAMs-related factors. **A** PD-L1 expression and M1-type TAMs-related CD molecules: CD64A, CD80 and CD86. **B** PD-L1 expression and M2-type TAMs-related CD molecules: CD163, CD200R and CD206. **C** PD-L1 expression and M1-type TAMs-related cytokines: IL-1α, IL-1β and TNF-α. **D** PD-L1 expression and M2-type TAMs-related cytokines: CSF1, IL-10 and TGF-β1

In addition, there is a positive correlation between PD-L1 expression and CD molecules associated with M1/M2-type TAMs including CD64A, CD80, CD86, CD163, CD200R and CD206. M1-type TAMs (Fig. 4A): CD64A (*r* = 0.4941, *p* < 0.0001), CD80 (*r* = 0.6242, *p* < 0.0001) and CD86 (*r* = 0.6047, *p* < 0.0001). M2-type TAMs (Fig. 4B): CD163 (*r* = 0.4374, *p* < 0.0001), CD200R (r = 0.4130, *p* < 0.0001) and CD206 (*r* = 0.2972, *p* < 0.0001). Moreover, PD-L1 expression was also positively correlated with cytokines related to M1/M2-type TAMs (Fig. 4C, D), including the cytokines of M1-type TAMs such as IL-1α (*r* = 0.2533, *p* < 0.0001), IL-1β (*r* = 0.3634, *p* < 0.0001), and TNF-α (*r* = 0.2886, *p* < 0.0001), as well as the cytokines such as CSF1 (*r* = 0.4478, *p* < 0.0001), IL-10 (*r* = 0.4305, *p* < 0.0001) and TGF-β1 (*r* = 0.2740, *p* < 0.0001) of M2-type TAMs. Unfortunately, PD-L1 expression was weakly correlated with most TAMs-elated cytokines. All the above results indicated that PD-L1 expression in TCGA-CESC data may be associated with M1/M2-type macrophage-related CD molecules and cytokines.

### Identification of induced differentiation of macrophages cells

**Fig. 5:**
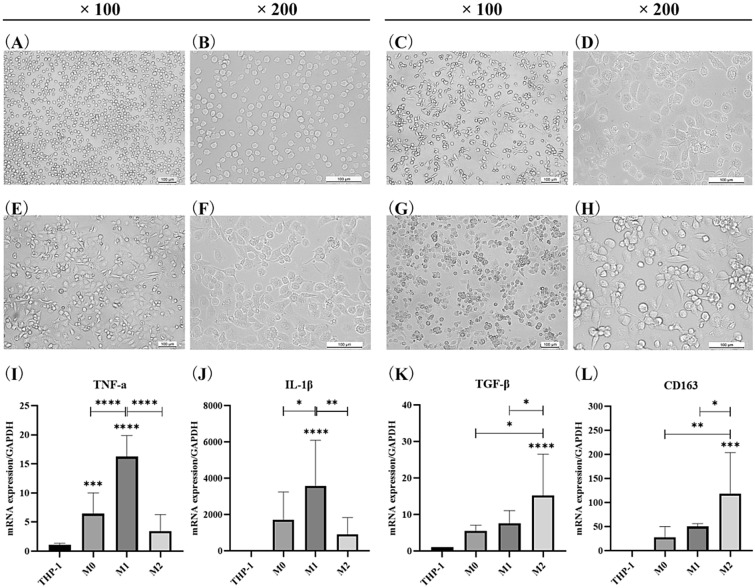
Morphological changes in induced differentiation of THP-1 cells and detection of specific marker mRNA. **A**, **B** THP-1 cells. **C**, **D** M0-type macrophages. **E**, **F** M1-type macrophages. **G**–**H** M2-type macrophages. **I** mRNA expression of TNF-α. **J** mRNA expression of IL-1β. **K** mRNA expression of TGF-β. **L** mRNA expression of CD163. *p < 0.05, **p < 0.01, ***p < 0.001, ****p < 0.0001

THP-1 cells were induced by LPS + IFN-γ and IL-4 + IL-13 to obtain M1-type and M2-ype macrophages, respectively. It was found that THP-1 cells were round or ovoid in morphology and suspended in the culture medium for growth (Fig. 5A, B). M0-type macrophages were induced by the addition of PMA, becoming spindle-shaped in morphology. A few cells appeared pseudopodia, and the cell size became larger (Fig. 5C, D). The morphology of cell was further changed after inducing with cytokines for 48 h. It was observed that the morphology of M1-type macrophages was mainly polygonal (Fig. 5E, F). M2-type macrophages were mostly round and short spindle-shaped. And some cells had protruding pseudopodia and were prone to aggregation and growth (Fig. 5G, H).

TNF-α and IL-1β were used as specific markers for M1-type macrophage, and TGF-β and CD163 were used as specific markers for M2-type macrophage. After differentiation induced by LPS and IFN-r, the expressions of TNF-α and IL-1β in M1-type macrophages were higher than those in M0-type macrophages (*p* < 0.0001, *p* = 0.0435) and those in M2-type macrophages (*p* < 0.0001, *p* = 0.0014), suggesting that M1-type macrophages was different from M0-type and M2-type macrophages (Fig. 5I, J). Similarly, after differentiation induced by IL-4 and IL-13, M2-type macrophages showed higher TGF-β and CD163 expression than M0-type macrophages (*p* = 0.0174, *p* = 0.0071) and M1-type macrophages (*p* = 0.0254, *p* = 0.0434), indicating that M2-type macrophages were different from M0-type and M1-type macrophages (Fig. 5K, L). To sum up, based on the specific molecules detection and morphological changes, we successfully established the M1/M2-type macrophage model.

### Effects of co-culture on tumor cell morphology and function

**Fig. 6:**
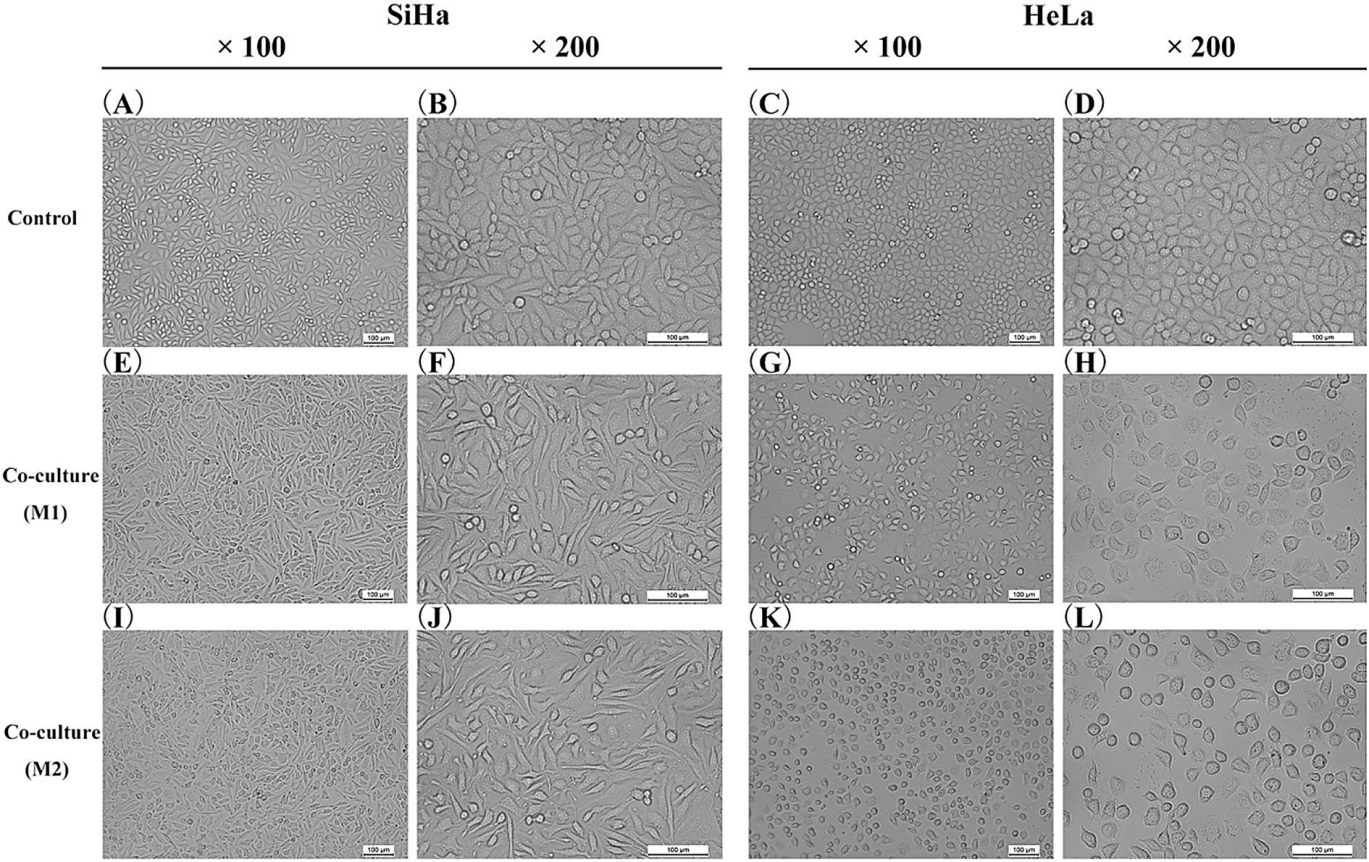
Morphological changes of cervical cancer cells after co-culturing with M1/M2-type macrophages. **A**, **B** Morphology of SiHa cell. **C**, **D** Morphology of HeLa cell. **E**, **F** The morphological change of SiHa cells after co-culturing with M1-type macrophages. **G**, **H** The morphological change of HeLa cells after co-culturing with M1-type macrophages. **I**, **J** The morphological change of SiHa cells after co-culturing with M2-type macrophages. **K**, **L** The morphological change of HeLa cells after co-culturing with M2-type macrophages

A co-culture system of CC cells and M1/M2-type macrophages was established through Transwell co-culture chamber. The results showed that the morphology of SiHa and HeLa cells changed greatly after co-culture with M1/M2-type macrophages. SiHa cells were blunt and round, with cobblestone-like arrangement and tight connections between cells (Fig. 6A, B). SiHa cells became a long spindle-shaped with disordered arrangement and wider intercellular space after co-culture, especially after co-culture with M2-type macrophages (Fig. 6E, F, I, J). HeLa cells were polygonal with good intercellular integration (Fig. 6C, D). After co-culture, the morphology of HeLa cells was irregularly round, and the degree of intercellular fusion was reduced, especially after co-culture with M2-type macrophages (Fig. 6G, H, K, L).

**Fig. 7:**
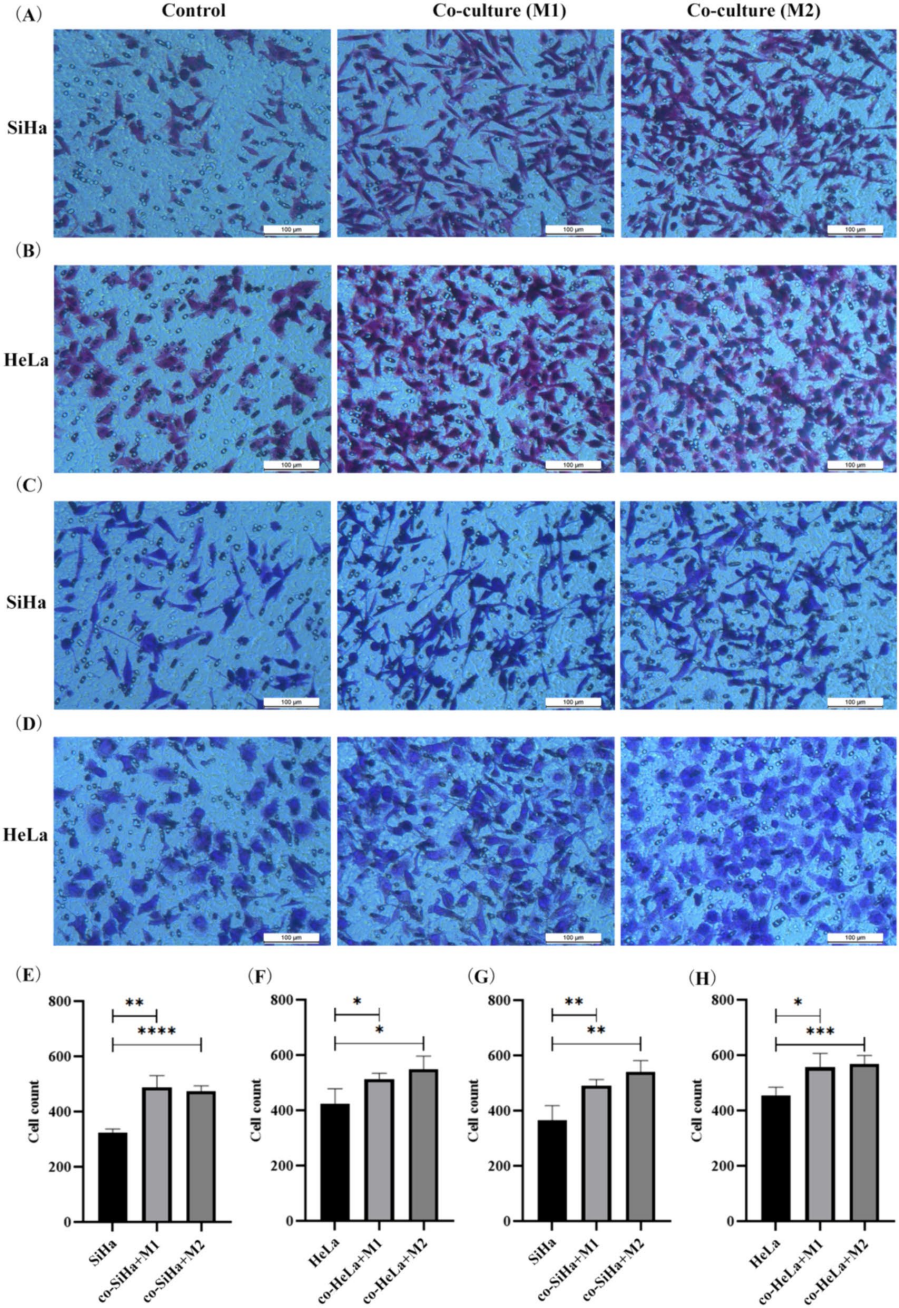
Effect of M1/M2-type macrophages on migration and invasion of cervical cancer cell. **A** Altered migration ability of SiHa cells after co-culturing with M1/M2-type macrophages. **B** Altered migration ability of HeLa cells after co-culturing with M1/M2-type macrophages. **C** Altered invasion ability of SiHa cells after co-culturing with M1/M2-type macrophages. **D** Altered invasion ability of HeLa cells after co-culturing with M1/M2-type macrophages. **E** Number of SiHa cell migration. **F** Number of HeLa cell migration. **G** Number of SiHa cell invasion. **H** Number of HeLa cell invasion. *p < 0.05, **p < 0.01, ***p < 0.001, ****p < 0.0001

SiHa and HeLa cells were co-cultured with M1/M2-type macrophages for 24 h in Transwell chamber (8 μM). The migration and invasion ability of CC cells were observed by counting and quantitative analysis of cells that passed through the membrane of micropore. As shown in Fig. 7, the number of SiHa cells passing through the chamber microporous membrane was significantly increased after co-culture, suggesting a significant enhancement in the migration and invasion ability of SiHa cells. M1-type macrophages enhanced the migration and invasion of SiHa cells (*p* = 0.0012, *p* = 0.0079). M2-type macrophages also play a similar role in SiHa cells (*p* < 0.0001, *p* = 0.0011). In addition, compared with the control group, the migration and invasion ability of HeLa CC cells could be improved by M1-type macrophages (*p* = 0.0396, *p* = 0.0183) and M2-type macrophages (*p* = 0.0121, *p* = 0.0008) after co-culture. In conclusion, the above results indicated that the migration and invasion ability of CC cells was enhanced after co-culture with M1/M2-type macrophages, especially after co-culture with M2-type macrophages.

### M1/M2-type macrophages can promote the upregulation of PD-L1 expression in CC cells

**Fig. 8:**
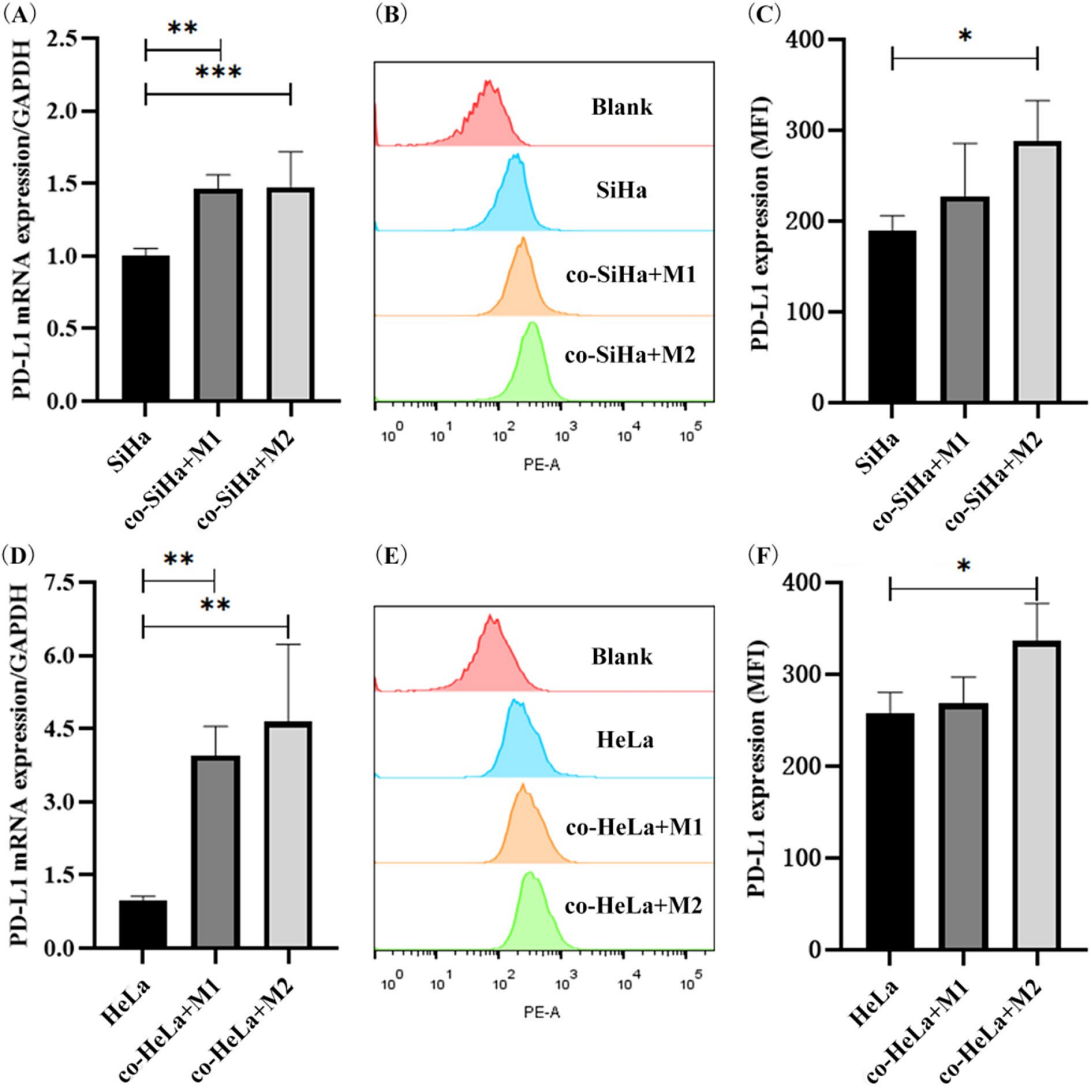
M1/M2-type macrophages can promote the expression of PD-L1 in cervical cancer cells. **A** The mRNA expression level of PD-L1 for SiHa after co-culturing with M1/M2-type macrophages. **B** Flow cytometry of PD-L1 expression in SiHa cells. **C** The protein expression level of PD-L1 for SiHa after co-culturing with M1/M2-type macrophages. **D** The mRNA expression level of PD-L1 for HeLa after co-culturing with M1/M2-type macrophages. **E** Flow cytometry of PD-L1 expression in HeLa cells. **F** The protein expression level of PD-L1 for HeLa after co-culturing with M1/M2-type macrophages. *p < 0.05, **p < 0.01, ***p < 0.001

SiHa and HeLa cells were collected after co-culture with M1/M2-type macrophages for the detection of mRNA and protein levels of PD-L1. The results showed that the mRNA level of PD-L1 on SiHa cells increased by about 1.5 times after co-culture with M1/M2-type macrophages (*p* = 0.0023, *p* = 0.0002, Fig. 8A). The mRNA expression level of PD-L1 on HeLa cells elevated about 4.0 times after co-culture with M1/M2-type macrophages (*p* = 0.0041, *p* = 0.0021, Fig. 8D). Meanwhile, after co-culture with M2-type macrophages, the expression of PD-L1 protein on the surface of SiHa and HeLa cells was increased by approximately 1.4 times (*p* = 0.0112, *p* = 0.0231, Fig. 8B, C, E, F). It can be seen that M1/M2-type macrophages can upregulate the mRNA and protein expression of PD-L1 in SiHa and HeLa cells. In brief, for M1/M2-type macrophages, there was a consistent effect on increasing the expression levels of PD-L1 mRNA and protein in SiHa cells, and a significant effect on promoting the expression of PD-L1-mRNA in HeLa cells. In addition, compared with M1-type macrophages, M2-type macrophages significantly promoted the expression of PD-L1 mRNA and protein in HeLa cells.

### M2-type macrophages promote PD-L1 expression in CC cells through the PI3K/AKT pathway

**Fig. 9:**
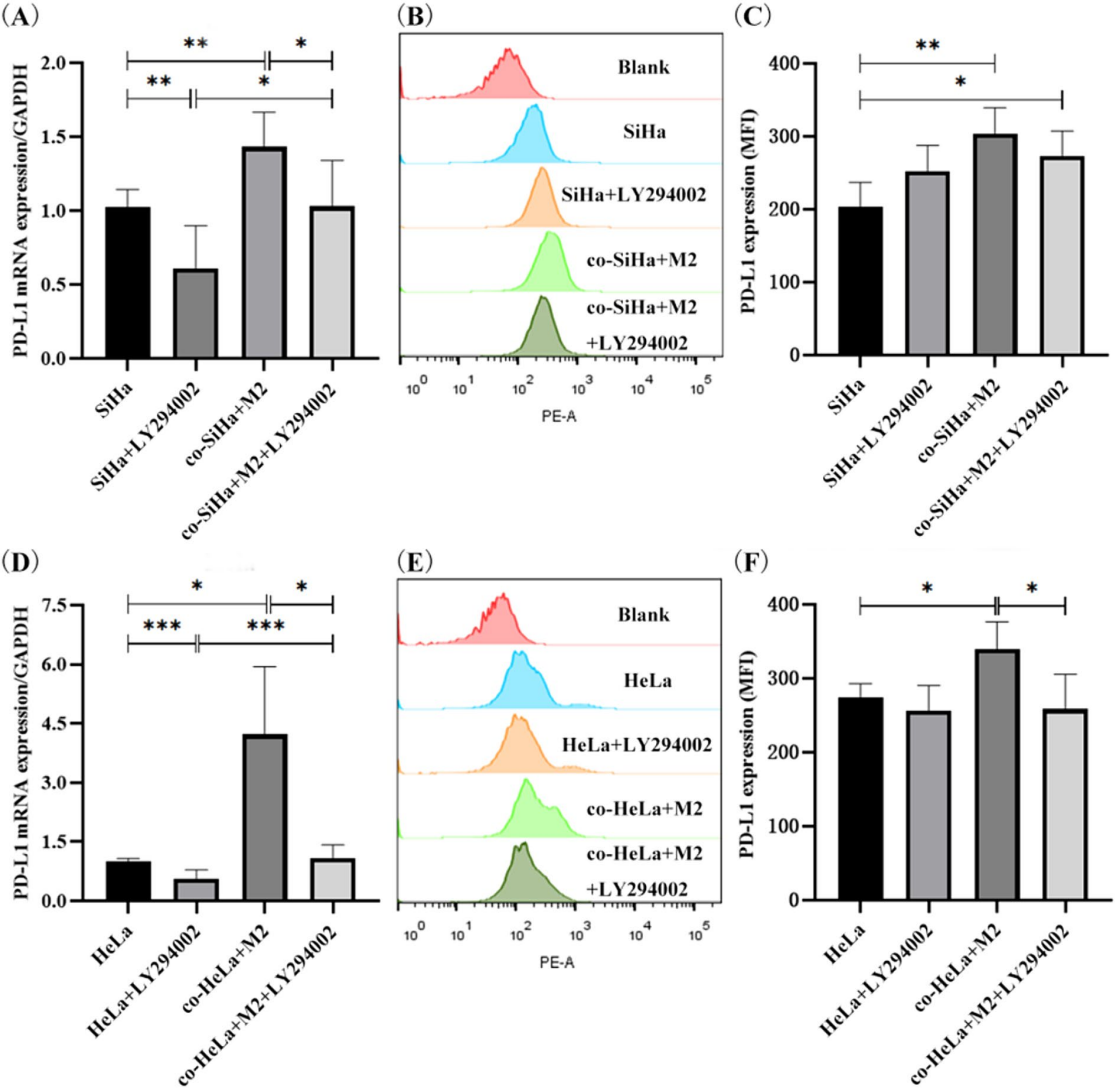
Effect of PI3K inhibitor on PD-L1 expression in cervical cancer cells. **A** The mRNA expression level of PD-L1 in SiHa cells. **B** Flow cytometry of PD-L1 expression in SiHa cells. **C** The protein expression level of PD-L1 in SiHa cells. **D** The mRNA expression level of PD-L1 in HeLa cells. **E** Flow cytometry of PD-L1 expression in HeLa cells. **F** The protein expression level of PD-L1 in HeLa cells. *p < 0.05, **p < 0.01, ***p < 0.001

PI3K inhibitor (LY294002) was added to observe the expression of PD-L1 in different groups. After pre-treatment with LY294002 for 1 h, SiHa and HeLa cells were co-cultured with M2-type macrophages. Compared with the control group (Fig. 9A, D), the mRNA expression level of PD-L1 in SiHa and HeLa cells was significantly decreased after adding LY294002 inhibitor (*p* = 0.0058, *p* = 0.0006). The mRNA level of PD-L1 in SiHa and HeLa cells was significantly increased after co-culture with M2-type macrophages (*p* = 0.0058, *p* = 0.0410), while the results were partially reversed after treatment with LY294002. The expression of PD-L1-mRNA was lower than that in co-culture group and close to the control group (*p* = 0.0140, *p* = 0.0410). It was also consistent in the protein expression of PD-L1 (Fig. 9B, C and E, F). The protein level of PD-L1 in SiHa and HeLa cells was upregulated after co-culture with M2-type macrophages (*p* = 0.0078, *p* = 0.0287). After being treated with LY294002, the promotion effect of M2-type macrophages on PD-L1 expression in SiHa and HeLa cells was partially reversed, that is, the protein expression of PD-L1 was lower than that in co-culture group (*p* = 0.5353, *p* = 0.0388). In conclusion, the PI3K inhibitor (LY294002) can reduce mRNA and protein expression of PD-L1 in SiHa and HeLa cells co-cultured with M2-type macrophages. Hence, M2-type macrophages may upregulate the PD-L1 expression of SiHa and HeLa cells through PI3K/AKT pathway.

## Discussion

CC is the fourth most common female cancer in the world. Currently, through vaccination and regular screening, the occurrence of CC can be effectively prevented. Therefore, the survival rate has been significantly improved through early detection and treatment. However, CC remains a killer in low- and middle-income areas for women who are partially advanced stage when diagnosed with limited treatment options due to limited resources [23]. Pembrolizumab approved by the FDA in 2018 has brought hope to patients with advanced CC and improved the curative effect of patients. But the patient response rate was lower than 30% [9], which was considered to be associated with immunosuppressive components of TME. TAM, as an immune cell, was involved in the formation of tumor suppressive microenvironment. Therefore, it will provide a new idea for improving patient efficacy and adopting combination therapy about exploring the regulatory mechanism of TAMs on PD-L1.

The expression of PD-L1 was gradually upregulated in normal cervix, cervical intraepithelial neoplasia and CC tissues. And 34.4–68.0% of PD-L1 positive expression was detected in tissues of CC patients [24, 25]. In our study, 52% of patients with CC showed positive expression of PD-L1 on tumor cells, and 45.9% of CC patients showed PD-L1-positive expression on stromal cells. Hence, it provided a potential therapeutic target for the use of PD-1/PD-L1 inhibitors because of the widespread expression of PD-L1 in tumor tissues. The expression range of PD-L1 was relatively wide due to the differences in patients' clinical characteristics, antibody sources, detection platforms, and evaluation criteria, so we believed that there was valid for the positive rate of PD-L1 in this study. Meanwhile, the expression of PD-L1 in CC tissues was related to fertility history, abortion history, differentiation grade, tumor size and FIGO stage in our study. Previous studies have shown that PD-1/PD-L1 pathway was closely associated with the history of fertility and abortion [26]. Pregnancy changed the immune status of the body, especially the uterine microenvironment including the cervix, while activating PD-1/PD-L1 pathways to induce and maintain immune tolerance. Moreover, PD-L1 expression tends to be higher in patients with advanced stage [27, 28], large tumor size [29], and poorly differentiated tumors. In summary, these results indicated that the variable efficacy of PD-1/PD-L1 inhibitor therapy may be due to individual differences and different degrees of disease development.

PD-L1 expressed on the surface of tumor cells can lead to T cell apoptosis and invalidation through specifically binding to PD-1 on the surface of tumor-infiltrating T cells, which is an important factor for tumor progression and poor prognosis. Previous studies have also shown that high expression of PD-L1 was associated with poor prognosis in various malignant tumors [30, 31]. In CC, the high expression of PD-L1 in tumor tissues was similarly related to poor prognosis [19, 32]. In our study, the positive expression of PD-L1 on tumor cells in CC tissue indicated a poor prognosis, while PD-L1 expression on stromal cells was not directly related to the prognosis of patients, suggesting that the expression of PD-L1 on tumor cells in CC TME was a reliable prognostic predictor.

The low response rate of PD-1/PD-L1 immunotherapy in patients with CC may be due to the body's self-resistance to PD-1/PD-L1 inhibitors [9], including the loss of new tumor antigens in mismatch repair function, T cell dysfunction caused by T cell exhaustion, and the immunosuppressive components in TME [33, 34]. TAMs, as one of the most important immune cells in TME, were generally believed to play a key role in regulating the immunosuppression of PD-1/PD-L1 [35, 36]. First of all, macrophages can phagocytose PD‑1 antibody. Arlauckas et al. observed that PD-1 antibodies could quickly and effectively bind PD-1^+^ tumor-infiltrating CD8^+^ T cells after administration. However, PD-1^−^ macrophages can capture PD-1 antibodies from the PD-1^+^CD8^+^T cell surface within minutes, limiting the reactivation of exhausted T cells and rendering them incapable of antitumor effects [37]. Furthermore, macrophages inhibited T cells reactivated by PD‑1 antibody to migrate to tumor islets. T cells must possess some abilities to eradicate tumor cells. On the one hand, they should efficiently accumulate and migrate to interact with tumor cells, on the other hand, T cells must respond adequately to tumor antigens and other activation signals [38]. Currently, immunotherapies mainly focus on restoring the dysfunction of T cells [39, 40], but lack of studies on promoting T cells to migrate to tumor nests. CD8^+^ T cells poorly migrated and infiltrated into tumor islets resulted from long-lasting interaction with macrophages [38]. Therefore, macrophages depletion may reactivate CD8^+^T cells to migrate and invade tumor islets, and improve the therapeutic effect of PD-1/PD-L1 inhibitors. Thirdly, TAMs are capable of secreting cytokines such as TGF-β, prostaglandin E2 (PGE2) and CCL18, which mediate tumor-promoting activity and immunosuppression in the TME. Cytokines secreted by TAMs including TGF-β have been found to affect PD-L1 expression [41]. In breast cancer and melanoma, TGF-β can induce the upregulation of PD-L1 in tumor cells and tumor-associated angiogenesis [16, 42]. Moreover, TGF-β derived from TAMs inhibited T cell activity through phosphorylating Smad2/3 protein and inhibiting mitochondrial respiration, thus promoting the formation of tumor immunosuppressive microenvironment [43, 44]. In addition, PGE2 upregulated PD-L1 expression in ovarian cancer cells via activating the PI3K-AKT-mTOR pathway to influence tumor growth [17], while reducing the infiltration of CD8^+^T cells [18]. Similarly, Wang et al.'s analysis showed that the upregulation of CCL18 secreted by M2-type macrophages can activate the PI3K-AKT pathway, thereby affecting tumor proliferation [45]. Therefore, the role of TAMs in the formation of tumor immunosuppressive microenvironment cannot be ignored. In short, macrophages play an important role in the treatment of PD-1/PD-L1. It will lay a theoretical foundation for improving the curative effect of combination therapy in patients by paying attention to the regulatory mechanism of TAMs on PD-1/PD-L1.

Inflammatory cells comprise a high proportion of the tumor context, among which macrophages, also known as TAMs, are one of the most abundant components in the TME [46]. As an important group of inflammatory cells in the TME, macrophages are well known for their plasticity and heterogeneity [47]. According to the different stimulating factors and secreted products, macrophages subpopulations are traditionally classified into classic activated macrophages (M1) and alternative activated macrophages (M2) [46]. However, on account of macrophages can express overlapping M1 and M2 genes, this nomenclature is too simple to label macrophages with M1 or M2 [48]. Macrophages perform pro-inflammatory and tumor suppressive functions under the exposure of pro-inflammatory stimuli including IFN-γ and lipopolysaccharide (LPS). Specifically, macrophages can contribute to antitumor immunity by releasing IL-1β, IL-12, IL-23, TNF-α and reactive nitrogen [13]. While in the TME, macrophages are induced to present the alternatively activated characteristics. High levels of IL-10 and the expression of mannose and galactose receptors which are secreted by macrophages are induced by anti-inflammatory stimuli including IL-4, IL-13 and immune complexes [49]. Such macrophages express high levels of Arg-1, TGF-β, and IL-10, which play an anti-inflammatory role and promote tumor cell proliferation, metastasis, angiogenesis and intravasation [13]. Under different signal stimulation, M1-type TAMs and M2-type TAMs play a dynamic change [50]. Therefore, the origin, the stimuli and the phenotype should be together to define macrophages [4]. In the study of Lai and Lv et al., LPS and IFN-γ were added to obtain M1-type TAMs, and M2-type TAMs were obtained by adding IL-4 and lL-13 [51, 52]. THP-1 cells were differentiated by exposure to PMA followed by addition of human recombinant IFN-γ + LPS and IL-4 to obtain M1-type and M2-type TAMs, respectively [53, 54]. Similarly, in our study, M1-type and M2-type TAMs were obtained by IFN-r + LPS and IL-4 + IL-13, respectively. The changes in cell morphology were consistent with the descriptions in previous study. And the characteristic markers of M1-type TAMs showed high expression of TNF-a and IL-1β, and M2-type TAMs highly expressed TGF-β and CD163, which further confirmed the M1/M2-type TAMs model obtained by induced differentiation. It laid a foundation for the subsequent cell experiments in vitro.

Through establishing the co-culture system of TAMs and tumor cells, the effect of TAMs on CC cells morphology was initially observed. There were significant changes in cell morphology after co-culturing with M1/M2-type TAMs, especially after co-culturing with M2-type TAMs. This was similar to the results of Chiu et al. and Ni et al. The morphology of oral squamous carcinoma cell was altered and intercellular tight junctions were disappeared after co-culturing with M2-type TAMs [55, 56]. In addition, the migration and invasion ability of tumor cells was enhanced by co-culturing with TAMs [57], which also reached a consistent conclusion in studies on gastric cancer cells and lung cancer cells [58, 59]. In our study, compared with the non-co-culture group, the migration and invasion ability of SiHa and HeLa cells were significantly enhanced in the co-culture group, especially after co-culture with M2-type TAMs. Hence, the above results indicated that TAMs can affect the morphology of CC cells, enhance the migration and invasion ability of CC cells, and lead to tumor progression, especially after co-culturing with M2-type TAMs.

There was a high correlation between the infiltration of TAMs and PD-L1 expression. CD68 is a marker for pan-macrophages. CD163 is considered as a specific molecular marker for M2-type TAMs. In the study of Deng et al., the density of CD68^+^ and CD163^+^ macrophages in PD-L1-positive nasopharyngeal carcinoma tissues was significantly higher than that in PD-L1-negative tissues [60]. Similarly, in the study of Harada et al., the intratumoral infiltration of CD68^+^ and CD163^+^ macrophages were significantly higher in gastric adenocarcinoma tissues with PD-L1-positive than in those PD-L1-negative [61]. In the study of Guo et al., it was reached a consistent conclusion in CC tissues. In this study, PD-L1 expression was positively correlated with the infiltration of CD68^+^ and CD163^+^ macrophages by performing IHC staining of 98 patients. Meanwhile, through GSVA and correlation analysis of TCGA-CESC data, it was found that PD-L1 was correlated with M1/M2-type TAMs and positively correlated with TAMs-related CD molecules and cytokines. And the correlation between M1-type macrophages and PD-L1 seems to be stronger than M2 macrophages in the TCGA-CESC data. Unfortunately, this is in conflict with the results of in vitro cell experiments, where M2-type macrophages have a more significant impact on tumor cells. There are several factors. Firstly, M1/M2-type macrophages were represented by characteristic molecules rather than actual macrophages in GSVA analyses. Therefore, there is a direct impact on the results for the selection of characteristic molecules. Secondly, in the correlation analysis between the expression of PD-L1 and CD molecules, we only selected representative TAMs-related CD molecules that were currently undisputed, resulting in contradictory results compared to in vitro experiments. In fact, different choices of CD molecules may result in different outcomes. In addition, PD-L1 expression was weakly correlated with most TAMs-elated cytokines, which should not be ignored that some cytokines were secreted to the periphery and did not exist in tissues. Furthermore, many of these cytokines share expression with other cells present in the inflammatory infiltrate of CC tissue, so the cytokines involved in our study are not specific and cannot determine the role of macrophages through such correlation analysis. Therefore, these findings provide preliminary support for a potential interaction between TAMs and PD-L1, which still needs to be further validated in practice.

At present, the combination therapy of PD-1/PD-L1 inhibitors and targeted TAMs has made certain achievements. The TGF-β inhibitors and colony-stimulating factor 1 receptor (CSF-1R) inhibitor were widely recognized targeted TAMs therapy [62, 63]. The combination therapy of TGF-β inhibitors (1D11 and galunisertib) and anti-PD-1/PD-L1 can upregulate the expression of immune response genes, restore the cytotoxic activity of T cells and antitumor activity of anti-PD-L1 [44, 64]. Tranilast, another TGF-β inhibitor, combined with anti-PD-1/PD-L1 can enhance the infiltration of M1-type TAMs in tumor tissues, thus improving the anti-PD-L1 efficacy [65]. CSF-1R inhibitor PLX3397 (pexidartinib) combined with PD-1 therapy showed a synergistic therapeutic effect in increasing the infiltration of CD8^+^T cells to improve antitumor efficacy [38, 66]. And it can also effectively reduce the occurrence of tumor neovascularization and ascites [67]. BLZ945 (CSF-1R inhibitor) can also effectively control tumor growth when combined with PD-1/PD-L1 blocking antibody [68, 69]. Therefore, the combination of targeted TAMs therapy with anti-PD-1/PD-L1 improves the efficacy of cancer treatment, while clearing the regulation of PD-L1 expression on tumor cells by TAMs is particularly important for combination therapy.

The regulatory mechanism of TAMs on PD-L1 has been reported in some solid tumors. Through co-culturing with tumor cells in vitro, M2-type TAMs could promote PD-L1 expression on breast cancer cells and esophageal cancer cells to affect tumor progress [16, 70]. Further, TAMs can induce PD-L1 expression on tumor cells through activating the PI3K/AKT pathway in ovarian cancer and lung cancer, thereby affecting tumor growth and drug efficacy, respectively [17, 18]. Therefore, tumor-infiltrating TAMs were involved in the regulation of PD-L1 on tumor cells via PI3K/AKT pathway. In our study, TAMs can increase the mRNA and protein levels of PD-L1 on CC cells. Moreover, M2-type TAMs can upregulate the expression of PD-L1 through the PI3K/AKT pathway. The effect of the PI3K/AKT pathway on immune cells and the immune microenvironment is complicated. PI3K/AKT molecules not only affect the expression of PD-L1 in cancer cells [71], but also induce the differentiation of macrophages into M2-type, thereby promoting tumor proliferation and reducing cell apoptosis. Specifically, in Wu et al. study, PI3K signal transduction upregulated HIF-1a expression to induce macrophage polarization, resulting in cancer cell migration, invasion and metastasis [72]. In addition, M2 macrophage polarization was induced via activation of the PI3K/AKT signaling pathway, which promoted CRC metastasis [73]. Therefore, on the one hand, TAMs induced the activation of PI3K/AKT signaling pathway in tumor cells to upregulate the expression of immune escape molecules; on the other hand, TAMs was influenced by PI3K/AKT pathway and its products to further promote tumor proliferation and metastasis.

TAMs and tumor cells are closely related in the TME. This study found that TAMs can affect tumor cell morphology, function and immune checkpoint expression, thereby exerting immunosuppressive effects, which may be partly responsible for resistance to PD-1/PD-L1 therapy. Therefore, this study provided a theoretical basis for the combination therapy of TAMs with PD-1/PD-L1 by exploring the mechanism for PD-L1 expression regulated by TAMs. However, the present study still has some shortcomings. Firstly, due to the limitation of paraffin-embedded specimens, the expression and correlation of PD-L1, CD68 and CD163 should be explored in a larger population cohort. Secondly, this study explored the effects of TAMs on the morphology, function and the expression of PD-L1 on tumor cells by constructing a co-culture system. But the relationship between alterations in cell morphology and function and PD-L1 expression was ignored. We could not determine whether the changes of cell morphology and function affected PD-L1 expression, or whether the expression of PD-L1 changed the cell morphology and function, or whether there was no relationship between them. It needs to be further explored. Thirdly, in this study, M2-type TAMs obviously increased PD-L1 expression of CC tumor cells, significantly changed the morphology of tumor cells, and increased their migration and invasion functions. Combined with previous studies, M2-type TAMs often acted as an adverse factor to promote tumor growth and often formed a tumor suppressive microenvironment that affected the treatment of PD-L1. Therefore, we only focused on the regulatory mechanism of M2-type TAMs on CC cells, while studies on the regulatory mechanism of M1-type TAMs on PD-L1 was lacking. Meanwhile, it was not comprehensive enough for the regulatory mechanism of PD-L1 expression on CC cells by M2-type TAMs, and was also lacking animal experiments. We will focus on using different methods to confirm the molecular changes of pathway in future research and establishing the animal model for in vivo experiments to improve the study.

## Conclusion

In conclusion, PD-L1 was positively expressed on tumor cells in 52% of CC patients, and could be used as a prognostic predictor, and was closely related to CD163^+^TAMs infiltration. In addition, TAMs can affect the morphology of CC cells and enhance cell migration and invasion ability. M2-type TAMs may upregulate the expression of PD-L1 on CC cells through PI3K/AKT pathway, thus affecting the progression of tumor. In conclusion, studying the regulatory mechanism of TAMs on PD-L1 expression in CC cells provided a theoretical basis for improving patient efficacy and combined therapy.

## Data Availability

All data generated or analyzed during this study are included in this published article.

## Abbreviations

CC: Cervical cancer
TME: Tumor microenvironment
PD-L1: Programmed cell death ligand 1
PD-1: Programmed cell death factor 1
ICIs: Immune checkpoint inhibitors
ORR: Objective response rate
IL: Interleukin
CSF-1: Colony-stimulating factor 1
Arg: Arginnse-1
PI3K/AKT: Phosphatidylinositol 3-Kinase/protein kinase B
IHC: Immunohistochemistry
qRT-PCR: Quantitative real-time PCR
GSVA: Gene setvariation analysis
IFN-γ: Interferon-r
TNF-α: Tumor necrosis factor α
TGF-β: Transforming growing factor β
CSF-1R: Colony-stimulating factor 1 receptor
LPS: Lipopolysaccharide
